# QbD Approach for the Development of Tea Tree Oil-Enhanced Microemulgel Loaded with Curcumin and Diclofenac for Rheumatoid Arthritis Treatment

**DOI:** 10.3390/gels10100634

**Published:** 2024-09-30

**Authors:** Shivam Pathak, Ruhi Singh, Afzal Hussain, Nasir Ali Siddiqui, Saurabh Mittal, Annie Gupta

**Affiliations:** 1 Department of Pharmaceutics, Amity Institute of Pharmacy, Amity University, Noida 201303, UP, India; shivam.kb.pathak@gmail.com (S.P.); ruhisingh1945@gmail.com (R.S.); 2 Department of Pharmacognosy, College of Pharmacy, King Saud University, Riyadh 11451, Saudi Arabia; afihussain@ksu.edu.sa (A.H.); nsiddiqui@ksu.edu.sa (N.A.S.); 3 Department of Medicinal Chemistry, Amity Institute of Pharmacy, Amity University, Noida 201303, UP, India

**Keywords:** central composite rotatable design, curcumin, diclofenac, microemulsion gel, QbD, tea tree oil

## Abstract

Rheumatoid arthritis, a chronic autoimmune disorder affecting millions worldwide each year, poses a significant threat due to its potential for progressive joint damage and debilitating pain if left untreated. Topical anti-inflammatory and analgesic treatments offer localized relief with reduced systemic side effects compared to conventional oral therapies, making them a promising option for managing rheumatoid arthritis. Therefore, the current study endeavored to formulate a microemulsion gel formulation loaded with diclofenac and curcumin for topical administration in the management of rheumatoid arthritis, utilizing Tea tree oil. The ratio of surfactant and cosurfactant was 4:1, assessed by pseudoternary phase diagram on the basis of the maximum emulsification region. The microemulsion underwent optimization using a Central Composite Rotatable Design (CCRD) with constraints of minimum particle size, polydispersity index, and maximum transmittance. The Curcufenac-T microemulsion had a particle size, polydispersity index (PDI), and transmittance of 151.82 ± 15.9 nm, 0.287 ± 0.021, and −5.78 ± 0.26 mV, respectively. DSC analyses confirmed the stability and compatibility of diclofenac and curcumin within the formulation. The microemulsion was changed into gel form by incorporating 1% carbopol-934. Skin permeation analysis revealed that the percentage of diclofenac permeated at 0.5 h from Curcufenac-T microemugel and the conventional gel was 12.1% and 3.9%, respectively, while at 12 h, the rates were 82.6% and 34.2%, respectively. In vitro permeability demonstrated significant potential for the effective delivery of diclofenac and curcumin to targeted sites, compared to conventional gel. Therefore, it was deduced that the Tea tree oil integrated diclofenac and curcumin microemulsion gel could enhance the effectiveness of diclofenac and serve as a promising vehicle for rheumatoid arthritis treatment.

## 1. Introduction

Rheumatoid arthritis (RA) is a persistent autoimmune condition distinguished by the inflammation of the synovium, resulting in joint discomfort, swelling, pain, and rigidity. It affects roughly 1% of the population worldwide, with a greater occurrence in females compared to males [1,2]. The pathology of RA entails the immune system erroneously targeting the body’s own tissues, resulting in damage to the synovium, cartilage, and bone within the joints [1,3]. Present treatment modalities for RA predominantly encompass nonsteroidal anti-inflammatory drugs (NSAIDs), disease-modifying antirheumatic drugs (DMARDs), and biologic agents [4,5,6,7]. The aforementioned medications can help manage symptoms and slow disease progression, but they often come with significant drawbacks, such as gastrointestinal side effects, immunosuppression, and the need for regular monitoring [8,9,10,11].

Diclofenac, a nonsteroidal anti-inflammatory drug (NSAID), is a monocarboxylic acid consisting of a phenylacetic acid derivative structure, has a molecular formula of C_14_H_11_Cl_2_NO_2_, a molecular weight of 296.15 g/mol, a melting point of 281.1 °C; it is poorly soluble in water but dissolves well in organic solvents such as ethanol and methanol. It is a potent drug commonly used in RA treatment and functions by blocking the activity of the enzyme cyclooxygenase (COX), consequently diminishing the production of prostaglandins responsible for inflammation and pain [4]. However, the long-term use of diclofenac through the oral route is associated with gastrointestinal complications [4,7,12]. In the current study, diclofenac is paired with curcumin. Curcumin, a natural polyphenol derived from the turmeric plant, is a natural compound that has attracted notice for its anti-inflammatory and antioxidant properties with a characteristic diketone structure, has a molecular formula of C_21_H_20_O_6_, a molecular weight of 368.39 g/mol, a melting point of 184.3 °C; it is practically insoluble in water but soluble in organic solvents such as ethanol, DMSO, and acetone.

Curcumin, a compound found in turmeric, and diclofenac, a nonsteroidal anti-inflammatory drug (NSAID), are used together in rheumatoid arthritis (RA) treatment for their complementary effects. Diclofenac reduces inflammation and pain by inhibiting cyclooxygenase enzymes (COX-1 and COX-2), which are involved in the inflammatory process [13,14]. Curcumin has demonstrated anti-inflammatory and antioxidant properties through various mechanisms, including the inhibition of inflammatory cytokines and oxidative stress. The combination of curcumin and diclofenac can enhance therapeutic efficacy by targeting different pathways involved in inflammation. Curcumin may help reduce the dose of diclofenac needed, potentially minimizing the side effects associated with higher doses of NSAIDs. Together, they offer a multifaceted approach to managing RA symptoms, potentially improving patient outcomes.

This study aims to address the limitations of current RA treatments by developing a novel microemulsion formulation containing diclofenac and curcumin for topical application. Microemulsions offer several advantages, such as improved solubility of drugs with low water solubility, improved drug penetration into the skin, and prolonged drug release [15,16]. Also, microemulsions are versatile and have the ability to incorporate a range of lipophilic and hydrophilic drugs [17,18,19,20]. The microemulsion is made up of 3 components: oil, water, and surfactant; instead of selecting any random oil, we selected Tea tree oil, which is of therapeutic importance in the treatment of RA. Tea tree oil harbors a potent constituent known as terpinen-4-ol, which effectively suppresses the lipopolysaccharide-triggered synthesis of inflammatory mediators such as the tumor necrosis factor-alpha (TNF-α) and interleukin-1beta (IL-1β) [21,22,23]. This natural ingredient complements the therapeutic effects of diclofenac and curcumin, enhancing the overall efficacy of the formulation [24]. By harnessing the synergistic effects of diclofenac, curcumin, and Tea tree oil within a microemulsion formulation, our study aims to develop a topical treatment for RA that offers superior efficacy, reduced side effects, and enhanced patient compliance.

## 2. Results and Discussion

### 2.1. Screening and Selection of Oils, Surfactant, and Cosurfactant

Tea tree oil was the oil of choice and was selected as the oil phase in order to develop synergetic anti-inflammatory and analgesic effects. Tea tree oil has the ability to modulate inflammatory pathways and penetrate the skin effectively [25,26], making it an ideal candidate for targeted drug delivery to inflamed joints, thereby augmenting the bioavailability and therapeutic effects of diclofenac and curcumin in the treatment of RA [27,28]. The oil of choice should possess the capacity to dissolve the requisite dosage of the drug effortlessly. Employing surfactants to enhance solubility may inadvertently trigger the precipitation of the drug over an extended duration. The calculated solubility of diclofenac and curcumin in Tea tree oil was 11.80 ± 0.85 and 9.60 ± 0.53 mg/mL, respectively.

Surfactants play a crucial role in reducing interfacial tension, which is vital for the formulation of microemulsions. Generally, nonionic surfactants are regarded as being less toxic compared to ionic surfactants [29,30]. The choice of surfactant was determined by considering factors such as the hydrophilic-lipophilic balance (HLB) value, solubility, and miscibility through comprehensive studies. The solubility study results revealed that diclofenac and curcumin showed the highest solubility in Tween 80, specifically 36.10 ± 2.14 mg/mL and 24.22 ± 1.34 mg/mL, respectively. Moreover, they exhibited excellent compatibility with Tea tree oil. Hence, Tween 80 was chosen as the surfactant for microemulsion formulation development. The cosurfactant functions by diminishing the interfacial tension and accomplishes this by occupying the empty voids within the surfactant molecule. The cosurfactant was chosen based on its compatibility with the selected oil and surfactant, as determined by miscibility studies. Transcutol-P demonstrated compatibility with both the chosen oil and surfactant and thus was selected. Transcutol-P serves as a penetration enhancer, rendering it an ideal component for facilitating drug delivery through the skin [31,32,33].

### 2.2. Selection of Smix Using Phase Titration Method

**Figure 1:**
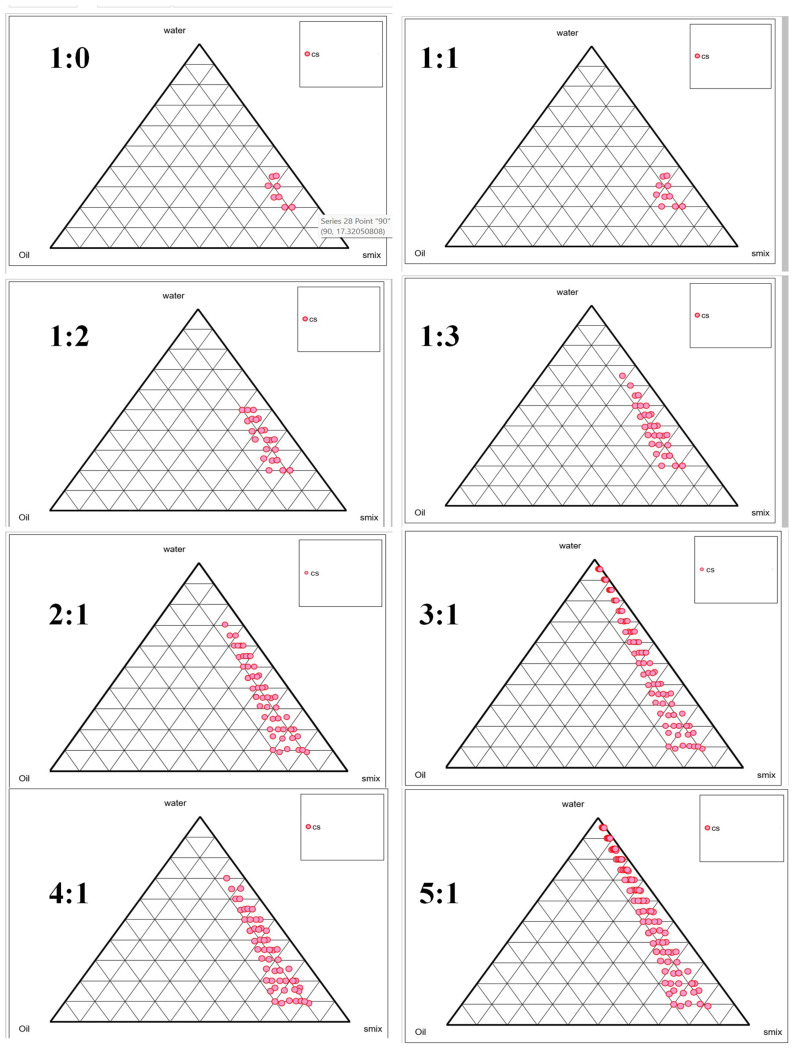
Pseudoternary phase diagrams constructed for various Smix ratios.

After initial solubility and emulsification efficacy tests, Tea tree oil was chosen as the oil phase, Tween 80 as the surfactant, and Transcutol-P as the cosurfactant for the microemulsion formulation. The optimal composition of the microemulsion system was determined using pseudoternary phase diagrams (Figure 1), a known method for delineating transparent, single-phase, low-viscosity microemulsions. These diagrams helped evaluate the Smix (Tween 80: Transcutol-P) volume ratios. Smix ratios of 1:0 had a smaller microemulsion area than those with 1:1, suggesting that a cosurfactant expanded the microemulsion region. This expansion was further intensified with 1:2 and 1:3. The microemulsion area increased significantly when the surfactant concentration was 2:1, 3:1, or 4:1 relative to the cosurfactant. As the ratio of Smix was elevated from 2:1 to 4:1., interfacial tension decreased due to the system’s elevated hydrophilic-lipophilic balance (HLB). Despite having a higher HLB value, a 5:1 Smix ratio had a smaller microemulsion area, suggesting that adding Tween 80 did not improve emulsification. Due to the Tween 80’s reduced fluidity, droplet size may be affected. Thus, the pseudoternary phase diagram analysis recommended a 4:1 (*v*/*v*) Smix ratio for microemulsions. Additionally, the HLB value of the 4:1 ratio of Smix was 12.8, which is in accordance with the present formulation. This is because, for the chosen Smix, the HLB value must surpass 10 when preparing an o/w type microemulsion [34]. 

### 2.3. Optimization of Nanoemulsion Using Central Composite Rotatable Design

PTSDs proved instrumental in determining the lowest and highest percentage of Smix and oil for the microemulsion formulation. Furthermore, because the microemulsion was of the oil/water type, it was selected from the PTSDs in the area where the oil concentration was lower than the water concentration; otherwise, it would have been water-in-oil. The range of oil taken was 2–4% based on the solubility of the drug and the amount of surfactant required. If the concentration of surfactant is less than 20%, it leads to the formation of nanoemulsion rather than microemulsion [32,35]. A Surfactant concentration of more than 20% gives a thermodynamically stable microemulsion. Based on this principle, the lower and upper limits of Smix selected were 20–25%. For the optimization of the microemulsion, CCRD design was chosen, and a total of 13 runs were generated, of which four were axial, four were factorial, and five were central runs, as shown in Table 1.

### 2.4. Data Analysis

The particle size (Y1) calculated had an experimental range between 44.3 and 65.9 nm, the PDI (Y2) ranged between 0.156 and 0.195, and the percentage transmittance (Y3) had an experimental range between 55.6 and 92.8%. Upon inputting the experimental values of particle size, PDI, and percent transmittance into the optimization software v23.1, polynomial quadratic models were suggested for the particle size and percent transmittance. For PDI, quadratic and linear models were suggested (Table 2).

### 2.5. Effect of the Independent Variable on the Particle Size

Microemulsion preparation relies significantly on its particle size since the size of the droplet determines drug release. It was intended to create a microemulsion with microscopic droplets. ANOVA revealed that the quadratic model had a higher R^2^ value (0.9919) compared to other models [36]. The equation below illustrates how the oil percentage (A) and mix percentage (B) affect the particle size.

Particle Size = 50.8 + 4.0472 × A − 6.70787 × B − 2.425 × AB + 1.33125 × A^2^ + 1.18125 × B^2^

**Figure 2:**
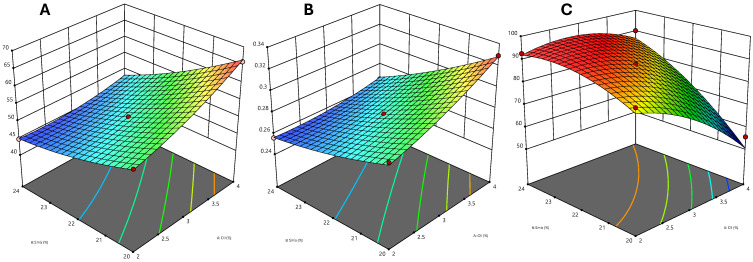
Response surface plots showing the effect of oil and Smix on (**A**) particle size, (**B**) PDI, and (**C**) percentage transmittance.

Based on this equation, the particle size was positively affected by the oil percentage (*p* < 0.0001) but negatively by the Smix percentage (*p* < 0.0001). The particle size shows an increase with rising oil concentration, whereas it decreases with an elevation in the Smix concentration. Figure 2A shows three-dimensional graphs for the particle size, which can help to understand this visually. The effect of the Smix concentration was higher compared to that of oil concentration on the particle size.

### 2.6. Effect of Independent Variable on PDI

Standard deviation divided by mean particle size is the measure of polydispersity. A more homogeneous particle size is achieved with a lower PDI. Using analysis of variance (ANOVA), we looked at the experimental data, and the summary statistics of the models revealed that quadratic models had higher R^2^ (0.9947)than linear model. All the droplet sizes were consistently within the specified range of the formulation’s polydispersity values, which were between 0.254 and 0.329. The equation below illustrates the influence of the independent variable on PDI.

PDI = 0.277 + 0.0137478 × A−0.0221798 × B − 0.0085 × AB + 0.00375 × A^2^ + 0.00325 × B^2^

Since the coefficient values of the independent variables were not statistically significant, they did not substantially affect PDI. On the other hand, PDI was negatively affected by Smix, but oil had a positive effect Figure 2B. This indicates that the PDI rises as the oil concentration rises and falls as the Smix concentration rises.

### 2.7. Effect of Independent Variable on Transmittance

The relationship between the droplet size and percentage transmittance serves as an indicator of the clarity of the microemulsion. A microemulsion’s transmittance increases as the size of its droplets decreases and vice versa. Upon analysis using ANOVA, the experimental outcomes indicated that the quadratic model exhibits a better R^2^ value of 0.9335 in the model summary statistics. A relationship between the percentage transmittance and independent variables is shown by the following equation:

Transmittance = 87.82 − 9.57793 × A + 11.1802 × B + 6.925 × AB − 5.16 × A^2^ − 4.535 × B^2^

The percentage of Smix (*p* < 0.0001) exhibited a positive impact on transmittance, while the percentage of oil had a negative impact (*p* = 0.0001). As the percentage of Smix increased, the percentage of transmittance also increased; however, as the percentage of oil increased, the percentage of transmittance decreased. The impact of the concentration of Smix was more pronounced in comparison to that of oil concentration on the percentage of transmittance. Figure 2C shows three-dimensional graphs for particle size, which can help to understand this visually.

### 2.8. Selection of Optimized Formulation

The selected parameters for the optimized microemulsion were minimum particle size and PDI, which indicates homogeneity, and a high transmittance, which is necessary for the stability of the formulation. Although oil had a therapeutic efficacy in the management of RA, it was taken to be in range as higher oil has been reported to show skin irritation in some cases. Surfactant concentrations in microemulsions promote enhanced solubilization of hydrophobic constituents, consequently bolstering the formulation’s stability and overall efficacy; thus, it was also in range. After careful examination, the software recommended the microemulsion having 2.35% oil and 24% Smix.

### 2.9. Physical Stability Studies of the Microemulsion

Performing physical stability testing on microemulsion formulations is essential for ensuring that they do not undergo any processes such as coalescence, separation of oil and water phase, crystallization of fat, transition in interfacial phases, precipitation, electrostatic or chemical interaction, etc. The optimized microemulsion was kinetically stable and exhibited no evidence of precipitation, breaking, or creaming when tested under a higher temperature range (continuous heating and cooling cycle) and also at low temperature, i.e., freeze-thaw and stress conditions like high shear using a centrifuge.

### 2.10. Characterization of the Optimized Microemulsion

#### 2.10.1. Particle Size, PDI, and Zeta Potential

**Figure 3:**
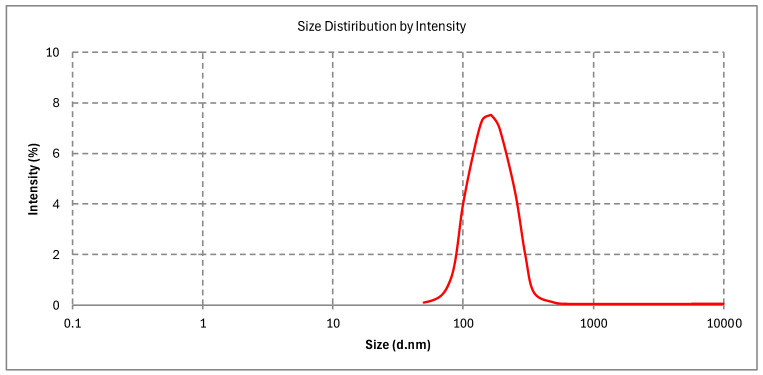
Particle size of Curcufenac-T.

**Figure 4:**
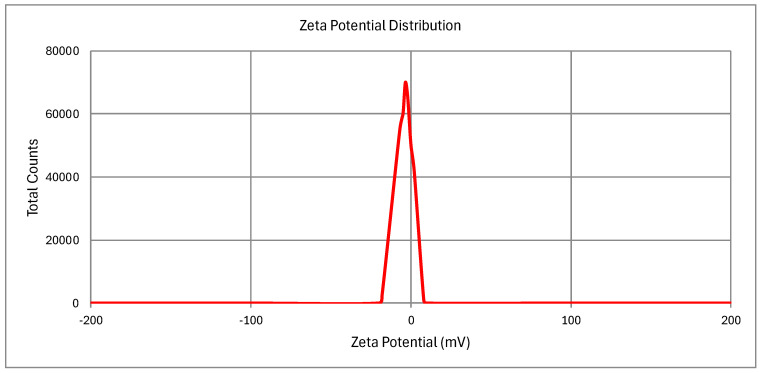
Zeta potential of Curcufenac-T.

The Curcufenac-T microemulsion (curcumin and diclofenac microemulsion with Tea tree oil) had a mean particle size of 151.82 ± 15.90 nm (Figure 3). The PDI values were determined to be 0.287 ± 0.021. It was consistent with the TEM findings that the microemulsion’s small particle size was caused by the reduced oil content. The microemulsion contained particles at the nanoscale, and research has indicated that the conversion to microemulsion gel does not impact the particle size. Figure 4 shows that the Curcufenac-T microemulsion’s zeta potential value was (−5.78 ± 0.26 mV). A little negative charge, which might appear because of the presence of free fatty acids or other ionic contaminants, is commonly seen when microemulsions are prepared with nonionic surfactants like Spans and Tweens, even though technically, there should not be any formation of surface charge [37]. As a result of acceptors of protons in the polar polyol groups forming hydrogen bonds with hydrogen atoms present on the surface of the aqueous medium, microemulsions are stabilized. Furthermore, the stability of the microemulsion made with the nonionic surfactant is enhanced by the steric effect produced by interactions between the hydrophobic oil phase and the tails of hydrocarbon chains [32,38].

#### 2.10.2. Percentage Transmittance, pH, and Refractive Index 

The Curcufenac-T microemulsion exhibited a percentage transmittance of 93.6 ± 0.45%. This suggests that the formulation was isotropic in nature, with high transmittance indicating particle stability and the absence of coagulation. The pH of Curcufenac-T microemulsion was measured at 5.44 ± 0.42. Additionally, the refractive index (RI) of Curcufenac-T microemulsion was determined to be 1.392 ± 0.011, indicating chemical stability and isotropy in the formulation [39].

#### 2.10.3. Surface Morphology

**Figure 5:**
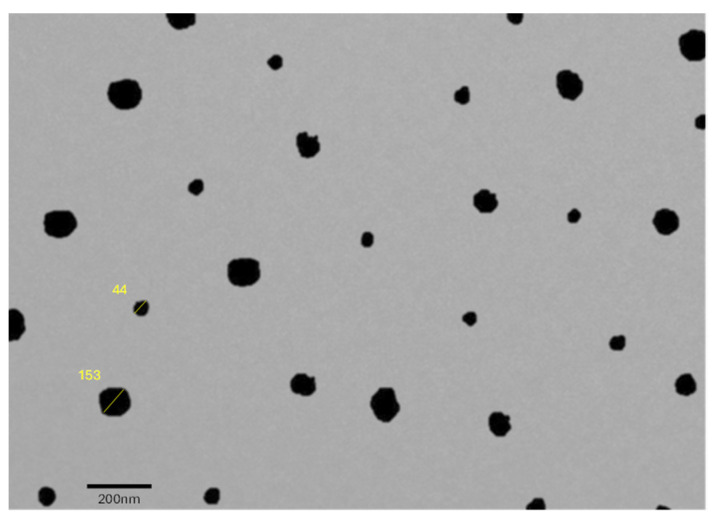
Morphological determination using TEM of Curcufenac-T.

The TEM picture of the optimized Curcufenac-T microemulsion depicted in Figure 5 reveals spherical nanostructured shapes. These results were consistent with the size of the particle determined through the light scattering methodology. The transmission electron microscopy (TEM) images mentions two distinct particle sizes of 44 nm and 153 nm. These measurements are consistent with the particle size analysis obtained using the light scattering method, confirming the reliability of both techniques.

### 2.11. Incorporation of Drug in Optimized Formulation

**Figure 6:**
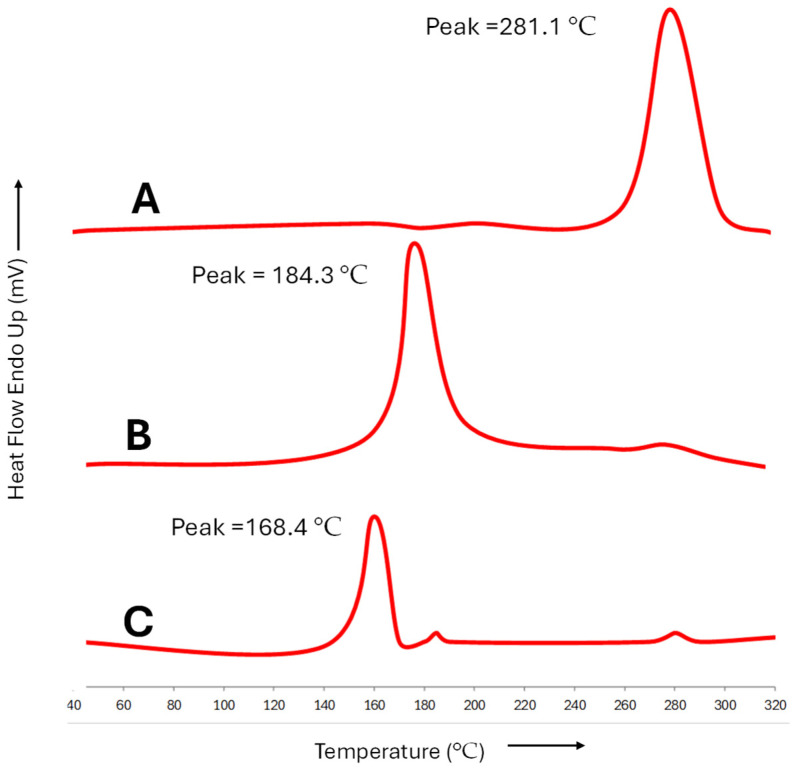
DSC of (**A**) diclofenac, (**B**) curcumin, and (**C**) lyophilized Curcufenac-T.

For the physical assessment of diclofenac and curcumin in the created microemulsions, the DSC thermogram was taken. According to the literature, diclofenac’s melting point is about 281.1 °C, and that of curcumin is 184.3 °C, which is close to the endothermic peak that the drug’s DSC spectra showed. In Figure 6, the lyophilized Curcufenac-T’s DSC thermogram displays a single peak of 168.4 °C. Mannitol, a cryoprotectant added to the microemulsion before lyophilization, was identified as the only peak in the differential scanning calorimetry (DSC) thermogram of lyophilized Curcufenac-T. Mannitol was selected as the cryoprotectant due to its favorable solubility in water of approximately 20% and its ability to crystallize upon freeze-drying [40,41,42]. Moreover, the distinct melting point of mannitol and the drug facilitated easy differentiation. Environmental and instrumental variables are among the many potential causes of variation in the peak value of mannitol. Diclofenac and curcumin do not precipitate or leach from their formulation since no peak was seen, suggesting molecular dispersion and the absence of a solid state [32,34,43].

### 2.12. Drug Content Uniformity of Optimized Gel

To assess the consistency of drug content in the gel, a drug content uniformity test was conducted. The results indicated no notable variance in drug content across three sections. The mean drug content of Curcufenac-T microemugel was found to be 96.4 ± 1.36%.

### 2.13. Skin Permeation Studies

**Figure 7:**
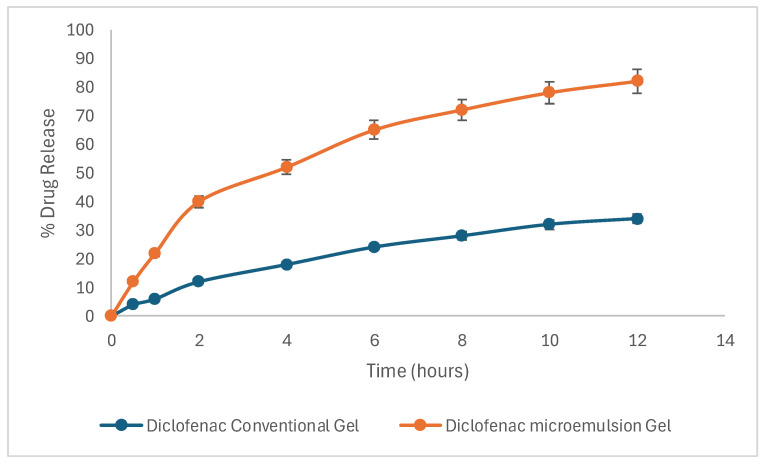
Drug release profile of diclofenac from conventional formulation and microemulsion gel.

The permeability of the Curcufenac-T microemugel and the conventional gel was assessed through permeation experiments. The rate of permeation (Js, μg/cm^2^/h) and time lag were ascertained by analyzing the slope and intercept of the drug permeation versus time plot under steady-state conditions [32,44]. The permeability coefficient (Kp) was ascertained through the division of the flux by the initial concentration of drug (C_0_) within the upper donor compartment. The analysis of permeation studies of skin revealed that the percentage of curcumin permeated at 0.5 h from Curcufenac-T microemugel and the conventional gel was 10.3% and 2.1%, respectively, while at 12 h, the rates were 74.2% and 2.5%, respectively. Skin permeation analysis revealed that the percentage of diclofenac permeated at 0.5 h from Curcufenac-T microemugel and the conventional gel were 12.1% and 3.9%, respectively, while at 12 h, the rates were 82.6% and 34.2%, respectively (Figure 7).

**Figure 8:**
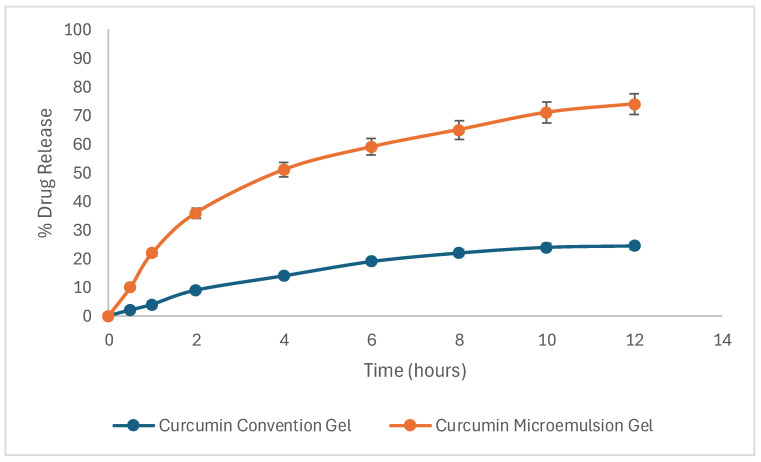
Drug release profile of curcumin from conventional formulation and microemulsion gel.

The flux of curcumin from Curcufenac-T microemugel and the conventional gel was determined to be 5.8 μg/cm^2^/h and 2.13 μg/cm^2^/h, respectively, with corresponding permeation coefficients of 11.6 × 10^−2^ cm/h and 4.26 × 10^−2^ cm/h, respectively (Figure 8). The flux of diclofenac from Curcufenac-T microemugel and the conventional gel was found to be 6.46 μg/cm^2^/h and 2.81 μg/cm^2^/h, respectively, with corresponding permeation coefficients of 0.1292 cm/h and 0.0562 cm/h (Figure 8).

Curcufenac-T microemugel exhibited a higher drug release and quick action in comparison to the conventional gel, indicating its superior permeation properties. In the context of topical delivery, the permeation coefficient is a crucial indicator of the efficacy of drug penetration into the stratum corneum. The heightened drug permeation observed with the microemulsion gel formulation can be attributed to several factors, including the enhanced solubility of the drug within the vehicle, efficient drug partitioning, and the disruptive effect exerted on the skin’s lipidic structure by the surfactants and cosurfactant components [45,46,47]. Moreover, the diminutive size of oil droplets within the microemulsion formulation microemulsion facilitated penetration through conventional intracellular and intercellular routes [32,38].

## 3. Conclusions

Our study presents a novel microemulsion designed to meet the therapeutic needs of RA patients. We aim to provide a complete therapeutic method that targets inflammation and pain by combining diclofenac, curcumin, and Tea tree oil in a topical delivery system. This will amplify effectiveness and reduce the risk of systemic side effects. The synergistic effects of diclofenac and curcumin are enhanced by the anti-inflammatory properties of Tea tree oil, specifically terpinen-4-ol. The use of surfactant (Tween 80) and cosurfactant (Transcutol-P) ensures the stability and optimal delivery of the active ingredients, improving the formulation’s bioavailability and therapeutic potential. By leveraging microemulsion technology, including improved drug solubility and skin penetration, our formulation offers a promising solution for RA management. Further preclinical and clinical investigations are warranted to validate the safety, efficacy, and long-term benefits of this microemulsion. Ultimately, our research aims to advance rheumatology by providing innovative treatment options that prioritize patient comfort, compliance, and quality of life for individuals with RA.

## 4. Materials and Methods

### 4.1. Materials

Diclofenac (CAS: 15307-86-5) and curcumin (CAS: 458-37-7) were sourced from CDH, Gujarat, India. Surfactant Tween 80 (CAS: 9005-65-6) was bought from S. D Fine Chemicals, New Delhi, India, and Transcutol-P (CAS: 111-90-0) was generously provided by Gattefosse India Pvt. Ltd. (Mumbai, India). Tea tree oil has been bought from Citspray Aroma Sciences in Maharashtra, India. Throughout this experiment, a variety of analytical-grade solvents and compounds were used to avoid any discrepancies.

### 4.2. Screening and Selection of Excipients

In the screening phase, a variety of oils, including mustard, garlic, clove, neem, and Tea tree, were evaluated for their suitability in formulating microemulsion. The surfactant choice was based on the solubility of diclofenac and curcumin, as well as the miscibility with oils. Similarly, the choice of cosurfactant was made by checking its compatibility with both the surfactant and the oil, ensuring optimal miscibility within the microemulsion system [43,44]. The goal of this stringent screening procedure was to determine which component combination would work best to create a microemulsion formulation with improved therapeutic potential for managing rheumatoid arthritis.

For assessing the solubility of diclofenac and curcumin, the following surfactants were employed: Tween 80, Span 80, Tween 20, and Solutol HS 15. Cosurfactants utilized to examine the miscibility of oils with surfactants included propylene glycol, sorbitol, Lauroglycol 90, Transcutol-P, and PEG 400. In summary, the drug was introduced into microcentrifuge tubes (MCTs) in abundant quantities along with 1 mL of surfactant, followed by continued agitation for 3 days at a temperature of 25 ± 1 °C to ascertain solubility. Additionally, for the miscibility evaluation, an equal volume of the solvent (either oil or surfactant) was mixed with 1 mL of cosurfactant, and the resulting solution’s clarity was assessed [32,48].

### 4.3. Smix Determination via the Phase Titration Technique

Pseudoternary phase diagrams (PTPDs) were constructed employing a combination of water, oil, and varying proportions of surfactant and cosurfactant (referred to as Smix) at ratios ranging from 1:0, 1:1, 1:2, 1:3, 2:1, 3:1, 4:1, to 5:1. The primary objective was to identify the optimal surfactant-to-cosurfactant proportion that would yield the maximum microemulsion region. PTPDs were made utilizing the method of aqueous titration, where the water phase was slowly titrated into the mixture under moderate stirring conditions for each oil and Smix ratio. Points where easily flowable and transparent microemulsions were achieved were designated as microemulsions and meticulously displayed on the phase diagram [32,43].

### 4.4. Experimental Design for Formulation Optimization

The microemulsion was optimized for QbD utilizing the response surface approach in the Design Expert^®^ program (version 8.0, Stat ease Inc., Minneapolis, MN, USA) [49]. Two of the most popular designs in the aforementioned strategy within the umbrella of response surface methodology (RSM), both the Central Composite Rotatable Design (CCRD) and the Box–Behnken design (BBD), serve as pivotal frameworks. They offer sophisticated strategies for exploring and optimizing complex response surfaces, facilitating the efficient analysis of intricate relationships between multiple variables. These are used most often in pharmaceutical research. Regarding response prediction, there have been reports that CCRD offers certain advantages over BBD. In contrast to the CCRD’s usage of two additional values at extremities (+α and −α) to fulfill the criterion of design for rotational stability, BBD used three points, representing a high, intermediate, and low value of independent variables [32,34]. Table 1 presents the results of the optimization of microemulsion based on the interaction of two independent variables: oil percentage and Smix percentage. The relationship is delineated, each exerting its influence on the outcome variables, which were particle size (Y1), polydispersity index (PDI) (Y2), and percent transmittance (Y3) (Table 3). Oil percentage and Smix percentage were the composition factors among these independent variables. The design itself created the intermediate and extremity values. According to the number of independent variables, CCRD recommended 13 random formulation runs, including four outcomes for factorial values, five for central values, and four for axial values. To make the optimal formulation, the following restrictions were applied: minimal size of the particle (nm), maximum PDI, and maximum percentage transmittance [43,44]. 

### 4.5. Physical Stability of Microemulsion

To assess the physical stability of the microemulsion, various tests were conducted. These included subjecting the microemulsion to freeze-thaw cycles, where it was alternately frozen at 20 °C for 24 h followed by thawing, repeating the process for a total of three cycles. Additionally, centrifugation studies were performed, involving the centrifugation of the microemulsion at 5000 rpm for 30 min to simulate stress conditions. Furthermore, the microemulsion underwent heating and cooling cycles, alternating between a temperature range of 4 °C and 40 °C for over six cycles. These rigorous tests were employed to evaluate the physical robustness and stability of microemulsion under various environmental conditions, ensuring its suitability for pharmaceutical applications [32,44].

### 4.6. Preparation and Analysis of Optimized Microemulsion

The microemulsion (Curcufenac-T) was created using Tea tree oil as an oil phase and purified water for a continuous water phase. Continuous vortexing was used at room temperature to dissolve 1% *w*/*v* diclofenac dimethylamine and 1% *w*/*v* curcumin in the oil phase. Once the drugs were dissolved in the oil, a 4:1 mixture of Tween 80 to Transcutol-P was added, followed by gradually adding Milli-Q water to this oily phase while continuously vortexing [34,43]. 

### 4.7. Particle Size, Zeta Potential, and PDI

Curcufenac-T microemulsion was studied using dynamic light scattering (Malvern Zetasizer, Nano S, Haryana, India) to determine their polydispersity index (PDI) as well as particle size. Adequate dilutions of the formulations were made utilizing deionized distilled water almost 100 times before examination. The scattering of light was recorded at room temperature and a 90° angle. Using the method of Laser Doppler Electrophoresis, the zeta potential was measured. Following a 200-fold dilution in deionized distilled water, a thorough mixing process, and triplicate analysis, the formulation was ready for each measurement [48].

### 4.8. Percentage Transmittance, pH, and Refractive Index

We determined the percentage transmittance of the undiluted mixture with the help of a UV spectrophotometer set at 630 nm and a blank of double-distilled water. At room temperature, the pH of the Curcufenac-T microemulsion was determined by employing a pH meter calibrated earlier. To find the refractive index, Abbe’s type refractometer was brought. An industry standard, castor oil, was used. Without diluting the formulation, three measurements of the refractive index were taken after a few drops were deposited on the slide [44,48].

### 4.9. Examining Surface Morphology via Transmission Electron Microscopy (TEM)

An analysis using TEM (Tecnai, G20, and Philips Scientific, Amsterdam, The Netherlands) was conducted to discern the morphological characteristics of the formulation. A single drop of a microemulsion, diluted 100 times, was carefully placed onto a copper grid that had been coated with a carbon layer. For the TEM analysis, the copper grid, adorned with the sample, was delicately immersed in a solution comprising 2% phosphotungstic acid, meticulously enhancing its visual contrast for subsequent microscopic analysis [34,48].

### 4.10. Examining the Integration of Diclofenac and Curcumin in Microemulsion: Insights from Differential Scanning Calorimetry (DSC)

Differential Scanning Calorimetry (DSC) testing was conducted on freeze-dried Curcufenac-T microemulsion to ascertain the proper distribution and integration of diclofenac and curcumin within the microemulsion matrix. Prior to DSC testing, the microemulsions were freeze-dried employing a cryoprotectant. Specifically, 10 mL of microemulsion having 5% (*w*/*v*) mannitol was subjected to a freeze-dryer (LaboGene, Lillerød, Denmark) at −70 °C for freeze-drying. In brief, a 4 mg freeze-dried sample was positioned in a pan and subjected to heating at a controlled rate of 20 °C per minute, spanning from 40 °C to 200 °C, under the continuous flow of inert nitrogen gas at a rate of 20 mL per minute. An empty pan was utilized as a reference for an accurate comparison [32].

### 4.11. Conversion of Microemulsion into Gel

To prepare the microemulsion gel, the method by Mittal et al. [32] for the conversion of the formulation into gel was used. Briefly, 1% (*w*/*v*) Carbopol-934 was dispersed in 100 mL of formulation using the sieving method and constant mixing with the assistance of a magnetic stirrer for 120 min. Once the dispersion process was completed, a suitable amount of triethanolamine (1000 μL) was introduced to the dispersion of carbopol. The stirring process was then maintained to create Curcufenac-T microemugel containing Tea tree oil, diclofenac, and curcumin with a uniform appearance [32]. 

### 4.12. Drug Content Uniformity and pH of Optimized Gel

The amount of drug in the microemulsion gel was determined by mixing 500 mg of Curcufenac-T microemugel in 5 mL of methyl alcohol. The mixture was vigorously mixed on a vortex mixer for 4 h to ensure the complete dissolution of the given drugs in methanol. The final solution was subsequently passed through a nylon filter having a pore size diameter of 0.22 μm. The samples were analyzed using a UV spectrophotometer to determine the concentrations of diclofenac and curcumin. Following appropriate dilution, absorbance measurements were taken at 285 nm for diclofenac and 420 nm for curcumin, with phosphate buffer (pH 6.8) used as a blank (Figure S1). The absorbance values obtained were inserted into the equations derived from previously prepared standard curves to calculate the concentrations of the drugs in the samples. 

The pH profile of the microemulsion gel was explored using a precalibrated pH meter, and the measurements were taken at room temperature. All measurements were performed in triplicate [48]. 

### 4.13. Skin Permeation Studies

Skin permeation experiments were conducted using freshly obtained caprine dermis sourced from a local abattoir. Skin preparation for permeation investigations involved several steps. At the outset, the fur on the animal’s skin was eliminated using a hair removal cream. Subsequently, the underlying tissue was operatively removed, leaving the dermis exposed. The dermis side was gently cleaned with isopropyl alcohol to eliminate any adhering fat. Following this, the skin was thoroughly washed with distilled water to ensure cleanliness. Finally, the prepared samples of skin were enveloped in aluminum foil and placed in a freezer at −20 °C until required [44,50]. 

To conduct the drug permeation study, a Franz diffusion cell was used. The lower receptor compartment was loaded with 10 mL of phosphate buffer solution with a pH of 6.8, which included 25% *v*/*v* of methanol for sustaining the sink condition [51,52]. The mixture was maintained at 37 ± 0.5 °C with continuous stirring at 100 rpm. After the skin with an uncovered surface area of 0.785 cm^2^ was left to stabilize for 4 h, the donor’s skin surface was treated with 500 mg of each sample (Curcufenac-T microemugel and conventional curcumin and diclofenac gel). At intervals of 0.5, 1, 2, 4, 6, 8, 10, and 12 h; 1000 μL of the sample was withdrawn from the receiver cell and immediately substituted with an equivalent volume of fresh medium. The samples were analyzed using a UV spectrophotometer to determine the concentrations of diclofenac and curcumin. Absorbance measurements were taken at 285 nm for diclofenac and 420 nm for curcumin, with phosphate buffer (pH 6.8) used as a blank. The absorbance values obtained were inserted into the equations derived from previously prepared standard curves to calculate the concentrations of the drugs in the samples [48]. 

## Figures and Tables

**Table 1:** Independent variables and response values.

Independent Variable			Response		
Run	Factor 1 A: Oil (%)	Factor 2 B: S_mix_ (*v*/*v*) (%)	Y1: Particle Size	Y2: % PDI	Y3: Transmittance
1	1.58579	22	47.6	0.264	90.2
2	2	20	53.7	0.286	85.7
3	2	24	44.8	0.256	92.6
4	3	19.1716	62.8	0.313	58.9
5	3	22	50.8	0.277	87.8
6	3	22	50.8	0.277	87.8
7	3	22	50.8	0.277	87.7
8	3	22	50.8	0.277	87.9
9	3	22	50.8	0.277	87.9
10	3	24.8284	44.3	0.254	92.8
11	4	20	65.9	0.329	55.6
12	4	24	47.3	0.265	90.2
13	4.41421	22	60.1	0.305	59

**Table 2:** ANOVA results for CCRD.

Source	Adjusted R2	Predicted R2	SD	CV %	Model Suggested
R1 (particle size)	0.9919	0.9664	0.6015	1.15	Quadratic
R2 (PDI)	0.9947	0.9778	0.0016	0.5766	Quadratic
R3 (% Transmittance)	0.9335	0.7244	3.57	4.37	Quadratic

**Table 3:** Independent and dependent variables utilized in CCRD: Constraints and ranges applied.

Independent Variable	Levels				
	Axial (−2)	Low (−1)	Medium (0)	High (+1)	Axial (+2)
A= Oil (%, *v*/*v*)	1.58	2	3	4	4.41
B = S_mix_ (%, *v*/*v*)	19.17	20	22	24	24.82
	Constraint			Importance	
Independent variable					
A = Oil (%, *v*/*v*)	In range			+++	
B = S_mix_ (%, *v*/*v*)	In range			+++	
Dependent variable				+++	
Y_1_ = Particle Size (nm)	Minimum			+++	
Y_2_ = PDI	Minimum			+++	
Y_3_ = Percentage Transmittance	Maximum			+++	

## Data Availability

The data presented in this study are openly available in article.

## Supplementary Materials

The following supporting information can be downloaded at: https://www.mdpi.com/article/10.3390/gels10100634/s1, Figure S1: UV Spectrum of (a) Diclofenac (b) curcumin (c) combination of diclofenac and curcumin.

## Abbreviations

COX	Cyclooxygenase
CCRD	Central Composite Rotatable Design
RA	Rheumatoid Arthritis
NSAID	Nonsteroidal Anti-Inflammatory Drug
PDI	Polydispersity Index
DSC	Differential Scanning Calorimetry
HLB	Hydrophilic-Lipophilic Balance
Smix	Surfactant and Cosurfactant Mixture
TNF-α	Tumor Necrosis Factor Alpha
IL-1β	Interleukin-1 Beta
NF-κB	Nuclear Factor Kappa B
TEM	Transmission Electron Microscopy
QbD	Quality by Design
DMARD	Disease-Modifying Antirheumatic Drugs
DMSO	Dimethyl Sulfoxide
RI	Refractive Index
TEM	Transmission Electron Microscopy
DSC	Differential Scanning Calorimetry
UV	Ultraviolet
MCT	Microcentrifuge Tubes
PTPD	Pseudo-Ternary Phase Diagram
BBD	Box–Behnken Design
RSM	Response Surface Methodology
PEG	Polyethylene Glycol
RPM	Revolutions Per Minute
