# Supplementary material for: QbD Approach for the Development of Tea Tree Oil-Enhanced Microemulgel Loaded with Curcumin and Diclofenac for Rheumatoid Arthritis Treatment
Source: Gels. 2024 Sep 30;10(10):634. doi: 10.3390/gels10100634 (PMC11507205; doi:10.3390/gels10100634)
Supplement: Supplementary file 1 [file gels-10-00634-s001.zip › gels-3201571 supplement.pdf]

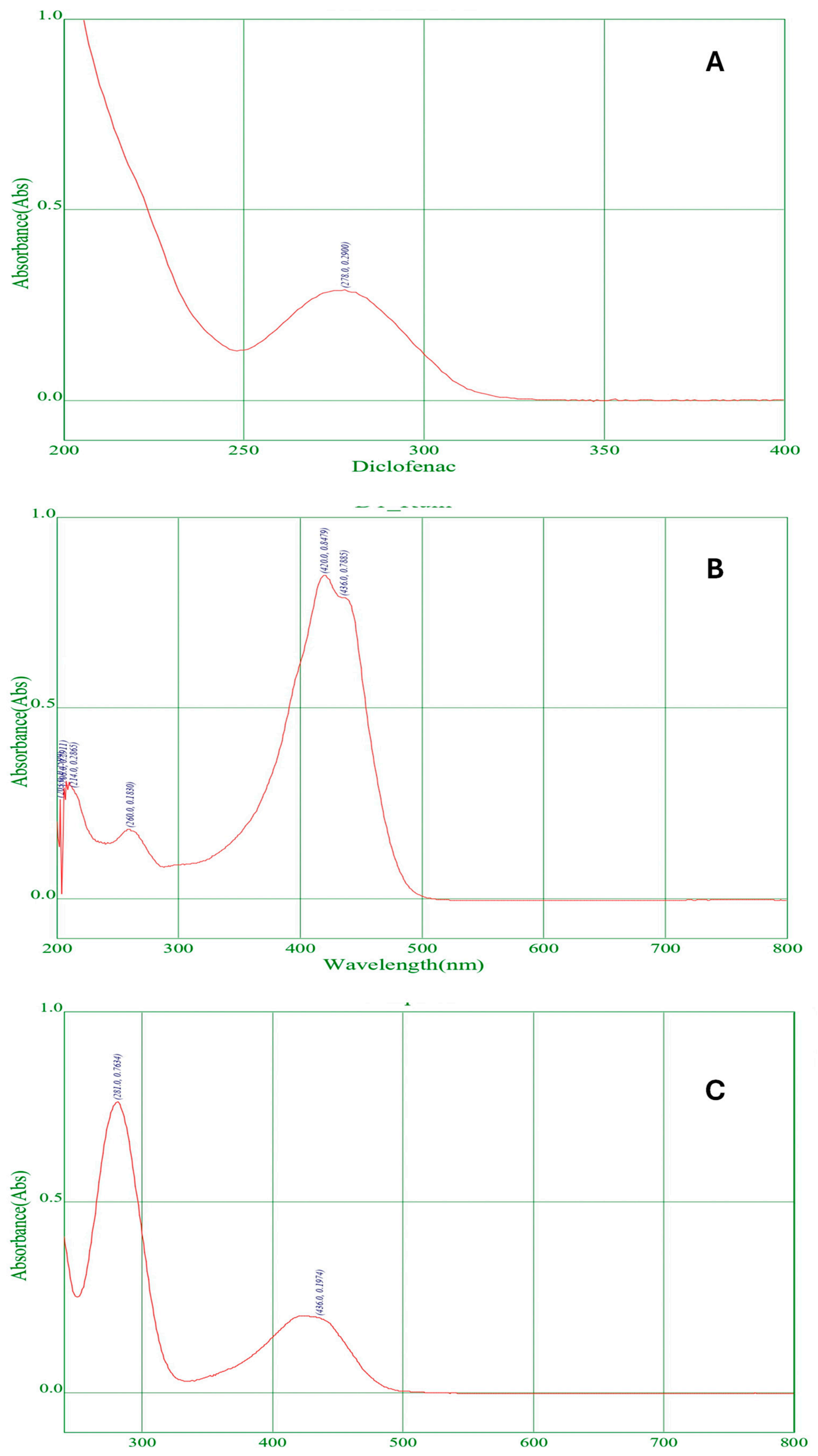

Figure S1: UV Spectrum of a) Diclofenac b) curcumin c) combination of diclofenac and curcumin

UV Absorption Profiles of Diclofenac and Curcumin

The UV spectral analysis of diclofenac and curcumin demonstrated their distinct absorption characteristics, evidenced by their respective λmax values: diclofenac peaked at 285 nm, while curcumin peaked at 420 nm. This pronounced separation in wavelengths indicated no overlap in their UV absorbance profiles.

In UV spectroscopy, the x-axis represented wavelength and the y-axis represented absorbance, producing distinct peaks for each compound. Diclofenac displayed a sharp peak at 285 nm, while curcumin exhibited a clear and separate peak at 420 nm. The absence of overlapping absorbance peaks was crucial for analytical applications, as it allowed for the accurate differentiation of these two substances during testing.

This separation was essential for maintaining the integrity of experiments involving diclofenac and curcumin, ensuring that their individual effects could be reliably assessed. Consequently, the results obtained from such analyses were both precise and dependable, facilitating effective evaluation of each compound's properties and interactions in various contexts.
